# Metagenomic analysis of the community structure and functional potential of *Tamarix* rhizosphere microbiomes along a soil salinity gradient

**DOI:** 10.3389/fmicb.2026.1756020

**Published:** 2026-03-04

**Authors:** Yanzhi Wang, Lijuan Zhang, Wei Huang, Ning Wang, Meng Sun, Longyuan Wu, Wei Wang, Chong Shi

**Affiliations:** 1 College of Resources and Environment, Xinjiang Agricultural University, Urumqi, China; 2 Institute of Microbiology, Xinjiang Academy of Agricultural Sciences/Xinjiang Laboratory Special Environmental Microbiology, Urumqi, China; 3 College of Grassland Science, Xinjiang Agricultural University, Urumqi, China

**Keywords:** functional adaptation, metagenomics, rhizosphere microorganisms, salt stress, *Tamarix*

## Abstract

**Introduction:**

Soil salinization strongly shapes rhizosphere microbial communities and their functional potential in arid ecosystems. *Tamarix* is a key halophytic shrub in desert saline–alkali environments, yet how its rhizosphere microbiomes respond to natural salinity gradients remains insufficiently understood. Here, we compared community structure, functional potential, and potential salt-adaptation strategies across a soil salinity gradient.

**Methods:**

Rhizosphere soils of *Tamarix* were collected from four sites (S1–S4) in Xinjiang, China spanning increasing salinity. Soil physicochemical properties were measured, followed by shotgun metagenomic sequencing. Taxonomic profiles and functional annotations were generated from metagenomic data and compared among salinity groups.

**Results:**

Salinity was associated with clear shifts in community composition. Bacteria dominated at low-to-moderate salinity, whereas archaeal relative abundance increased at higher salinity, with Euryarchaeota becoming dominant in the high-salinity group. Functional profiling indicated that core metabolic pathways remained prevalent along the gradient, suggesting relative stability in overall metabolic capacity. However, higher salinity was accompanied by enrichment of functions linked to genetic information processing (e.g., translation and replication/repair) and ion transport, while lipid metabolism, cell motility, and signal transduction were reduced.

**Discussion:**

Together, these results support a salinity-driven transition in microbial functional strategy from “growth expansion” toward “homeostasis maintenance.” Under high salinity, microbes appear to allocate more resources to maintaining cellular integrity and coping with stress, consistent with the observed enrichment of genetic information processing and repair-related functions. Mechanistically, the increased representation of Na^+^/H^+^ antiporter systems and V/A-type ATPases in the very high salinity group suggests that energy-dependent ion homeostasis is a prominent adaptation, helping regulate intracellular ion balance and mitigate salt toxicity. In contrast, pathways for compatible solute synthesis (e.g., betaine and ectoine biosynthesis) were relatively reduced, indicating that osmoprotection may rely less on de novo solute production and more on ion regulation and maintenance processes along this gradient. Overall, the metagenomic evidence clarifies how *Tamarix* rhizosphere microbiomes restructure taxonomically and functionally with increasing salinity and highlights key candidate mechanisms underpinning salt-stress adaptation. These insights provide a microbial basis for understanding plant–microbe interactions in desert saline–alkali soils and may inform ecological restoration and management in salinized regions.

## 1 Introduction

Soil salinization is recognized as one of the primary drivers of land degradation in arid and semi-arid regions worldwide, and agricultural productivity and ecosystem stability are consequently threatened (Ahmadi et al., 2022). It is estimated that more than 900 million hectares of land worldwide have been affected by soil salinization to varying degrees, and this area continues to expand; in China, saline–alkali soils cover approximately 99.13 million hectares, accounting for more than one tenth of the global saline–alkali land area (Yue et al., 2020; FAO, 2015). As a typical degraded soil type, saline soils are characterized by high salt content and a strong tendency to crust, which severely suppress biological growth, reduce species diversity, disrupt soil structure, weaken ecosystem functioning, and accelerate land degradation and desertification (Wang J. et al., 2025; Zhang et al., 2023). Among these plants, *Tamarix* species, which are dominant woody plants in arid and semi-arid regions, exhibit exceptional tolerance to salinity and drought and play a crucial role in maintaining ecosystem stability, improving soil structure, preventing wind erosion and sand encroachment, and promoting vegetation restoration (Liu, 2021).

Soil salinization not only reduces plant productivity but also fundamentally disrupts key microbially mediated processes, and is regarded as an important mechanism underlying declines in soil health and ecosystem functioning (Khamidov et al., 2022). Numerous studies have demonstrated that salt accumulation alters soil osmotic potential, ionic composition, and physicochemical conditions, by which microbial activity is inhibited and microbial diversity and functional attributes are reshaped, thereby affecting multiple functional pathways, including organic matter decomposition, nutrient cycling, and the maintenance of soil structure (Yan et al., 2022; Paul et al., 2024; Quiroga-González et al., 2025). Reviews have further indicated that the impacts of salinization on soil microorganisms share common features across ecosystems; the most frequently reported patterns include reduced microbial diversity, a community shift toward salt-tolerant taxa, and a redistribution of functions associated with biogeochemical processes (Li Y. et al., 2024; Li et al., 2021). Therefore, microbial responses to increasing salinity should be examined from both community composition and functional potential, which provides a key entry point for understanding process-level changes in saline soils (Zhu et al., 2025).

The effects of salinity on soil microbial communities exhibit a tightly coupled “structure–function” response: community composition and assembly are reconfigured, while functional potential and the underlying molecular adaptation mechanisms are systematically adjusted (Sun et al., 2022). Mechanistically, the response is first manifested as strong environmental filtering: elevated osmotic pressure and Na^+^/Cl^−^ toxicity constrain the growth and establishment of salt-sensitive taxa, causing community composition to shift toward salt-tolerant/halophilic lineages (Nguyen et al., 2020). This shift is often accompanied by reduced α-diversity and enhanced between-community differentiation (Cong et al., 2025). From the perspective of community assembly theory, increasing salinity typically increases the relative importance of deterministic processes, such that stronger niche constraints and more pronounced structural reorganization are observed under high-salinity conditions (Liu et al., 2022; Zhang et al., 2019). In parallel, salinity stress can drive a reallocation of functional investment (Du et al., 2023). Additional energy is required to maintain osmotic balance and ionic homeostasis, and salinity tolerance is enhanced via two osmoadaptation strategies: (i) “salt-out,” through compatible-solute synthesis and/or uptake; and (ii) “salt-in,” through inorganic ion accumulation (Li Y. et al., 2024; Long et al., 2018). At the genetic level, enrichment is frequently observed for modules associated with K^+^ uptake (Trk/Ktr systems), Na^+^ extrusion and homeostasis (Na^+^/H^+^ antiporters), and stress-mitigation functions such as protein chaperones, DNA repair, and ROS detoxification/scavenging (Gao et al., 2024). Overall, salinity does not merely “reduce microbial abundance”; rather, community assembly pathways and dominant lineages are altered, while salinity-related functions and genetic systems are concurrently reinforced, resulting in interpretable and testable reconfiguration of both community structure and functional potential along salinity gradients (Li et al., 2025).

Research on plant rhizosphere microbiomes has advanced considerably in recent years, comparatively little attention has been paid to the rhizosphere microbial communities associated with *Tamarix* in desert ecosystems (Tacca et al., 2024; Philippot et al., 2013). Existing studies have mainly focused on the ecophysiological traits of *Tamarix* or on the isolation and application of individual functional microorganisms, whereas the community composition, functional differentiation, and molecular adaptation mechanisms of *Tamarix* rhizosphere microbiomes remain largely unexplored (Xia et al., 2017; Liang et al., 2025; Osman et al., 2018). Therefore, in this study, metagenomic approaches were employed to perform a systematic analysis of rhizosphere soil samples collected from *Tamarix* stands in different regions, with the following specific objectives: (1) to characterize the composition and diversity of *Tamarix* rhizosphere microbial communities; (2) to elucidate the functional gene repertoire of the rhizosphere microbiome; (3) to investigate the relationships between microbial communities and environmental factors; and (4) discuss the salt adaptations of microorganisms to salt stress. By addressing these objectives, this study aims to clarify the ecological strategies by which *Tamarix* rhizosphere microbiomes adapt to saline–alkali stress and to provide a theoretical basis and technical support for understanding plant–microbe interactions and promoting the restoration of salinized desert ecosystems.

## 2 Materials and methods

### 2.1 Sampling site description and soil collection procedures

Sampling for this study was conducted in October 2024 in Xinjiang, northwestern China. Xinjiang is located in the inland region of northwestern China and is characterized by scarce precipitation, an arid climate, and pronounced soil salinization. Four sampling sites were established in Aksu Prefecture and Bayingolin Mongol Autonomous Prefecture, where rhizosphere soil samples associated with *Tamarix* were collected. To minimize the influence of rainfall on soil physicochemical properties and microbial communities, sampling was performed only after at least 3 consecutive days without precipitation. At each sampling site, healthy *Tamarix* individuals were selected, and three rhizosphere soil samples were collected as biological replicates (Zheng et al., 2021; Venturini et al., 2022; Xiao et al., 2021). The geographic coordinates and habitat characteristics of the sampling sites are summarized in Table 1.

**Table 1:** Sampling information for the *Tamarix* rhizosphere soil samples.

**Name**	**Sampling site**	**Longitude (E)**	**Latitude (N)**	**Elevation (m)**	**Description of sampling habitat**
S1	Wetland Nature Reserve, Yuli County, Xinjiang	86°19′52″	41°03′36″	825.3	*Tamarix* rhizosphere soil in wetland reserve
S2	Populus euphratica Park, Luntai County, Xinjiang	84°37′38″	41°23′78″	869.1	*Tamarix* rhizosphere soil in Populus euphratica park
S3	139 “Secret” Scenic Road, Aksu, Xinjiang	82°29′97″	40°89′94″	920.8	Roadside *Tamarix* rhizosphere soil
S4	Roadside saline–alkali riverbank, Luntai County, Xinjiang	84°27′10″	41°50′20″	862.5	Saline–alkali *Tamarix* rhizosphere soil

During sampling, a sterile spatula was first used to gently remove loose surface soil around the roots, after which the soil tightly adhering to the root surface was collected and visible stones and plant residues were removed. The collected soil samples were placed in sterile zip-lock polyethylene bags and sealed. All samples were transported to the laboratory within 24 h of collection under refrigerated conditions with ice packs. Upon arrival at the laboratory, each sample was divided into two subsamples: one portion was placed in plastic bags, air-dried at room temperature, and used for the analysis of soil physicochemical properties, while the other portion was immediately stored at −80 °C in an ultra-low-temperature freezer and subsequently submitted to Beijing Novogene Bioinformatics Technology Co., Ltd. (Beijing, China) for shotgun metagenomic sequencing.

### 2.2 Determination of soil physicochemical properties

To systematically characterize the physicochemical properties of soils from different sampling sites, the following indices were measured for each sample according to the *Soil Agrochemical Analysis Methods* and *the Handbook of Soil Agrochemical Analysis* (Bao, 2000; Lao, 1998). Soil organic matter (SOM) content was determined colorimetrically. Total nitrogen (TN) was measured using the Kjeldahl method. Total potassium (TK) was quantified with a flame photometer. Total phosphorus (TP) was determined by the molybdenum-antimony colorimetric method. Water-soluble total salts (Salt) in the soil were measured gravimetrically. Soil electrical conductivity (EC) was determined using a conductivity meter in soil-water suspensions prepared at a 1:5 (w/v) ratio. Soil pH was measured with a pH meter in soil-water suspensions prepared at a 1:2.5 (w/v) ratio.

### 2.3 Soil genomic DNA extraction

Approximately 0.5 g of rhizosphere soil was collected from each sample, and total genomic DNA was extracted using a soil DNA extraction kit (D2600, Solarbio, Beijing, China) according to the manufacturer's instructions. DNA concentration and purity were measured using a NanoDrop spectrophotometer and a Qubit fluorometer, with A260/280 and A260/230 ratios recorded; DNA integrity was assessed by agarose gel electrophoresis. Qualified DNA was used for metagenomic library construction. For each sample, 1 μg DNA was sheared to ~350 bp fragments using Covaris ultrasonication, followed by end repair, A-tailing, adapter ligation, purification, and PCR amplification to complete library preparation. Library fragment size was examined using AATI, and the effective library concentration was quantified by qPCR (>3 nM). Qualified libraries were pooled equimolarly according to the targeted sequencing output and sequenced on an Illumina platform using paired-end 150-bp (PE150) chemistry (Novogene Co., Ltd., Beijing, China).

### 2.4 Bioinformatic analyses

Raw sequencing reads generated on the Illumina NovaSeq platform were quality-filtered and adapter-trimmed using fastp (GitHub, 2022), yielding clean reads. Clean reads were assembled using MEGAHIT to generate scaffolds, which were split at ambiguous (N) positions to obtain N-free scaftigs (Qin et al., 2010; Li et al., 2015). For each sample, scaftigs (≥500 bp) were subjected to ORF prediction using MetaGeneMark (2010), and ORFs shorter than 100 nt were removed (Mende et al., 2012). Predicted ORFs were clustered using CD-HIT (2006) to remove redundancy, generating a non-redundant gene catalog (unigenes). Clean reads from each sample were aligned to the gene catalog using Bowtie2 to obtain per-gene read counts. Gene abundance was estimated by normalizing counts by gene length for downstream taxonomic and functional profiling. Genes with ≤ 2 mapped reads per sample were filtered, resulting in the final gene set.

Unigenes were searched against the Micro_NR (National Library of Medicine, 2025) database using DIAMOND (GitHub, 2026). Micro_NR was constructed by extracting bacterial, fungal, archaeal, and viral sequences from the NCBI NR database. Taxonomic assignments were determined using a lowest common ancestor (LCA) approach, and abundances and gene counts were summarized across taxonomic ranks (kingdom to species). Based on the taxonomic abundance matrix, Krona plots, relative-abundance profiles, and clustered heatmaps were generated. Multivariate ordination, including PCA, PCoA (ade4), and NMDS (vegan), was performed in R.

Unigenes were aligned to the KEGG (2026), eggNOG (2019), and CAZy (2014) databases using DIAMOND, and the top-scoring hit for each sequence was retained for annotation and downstream analyses. Functional abundances at different hierarchical levels (e.g., KEGG Level 2/3) were quantified based on functional annotations and the gene-abundance table. Functional composition was visualized, and clustering and ordination analyses (PCA/NMDS) were conducted. Between-group differences were evaluated using ANOSIM, and metagenomeSeq and LEfSe were applied to identify differentially enriched functions and taxa.

### 2.5 Statistical analyses

Species abundance heatmaps, relative-abundance plots, and principal coordinates analysis (PCoA) ordination plots were generated in R (v4.2.0; R Foundation for Statistical Computing, Vienna, Austria). Redundancy analysis (RDA) integrating soil physicochemical properties with community structure and functional profiles was performed using Canoco (v5.1; Microcomputer Power, Ithaca, NY, USA). Spearman's rank correlations between soil physicochemical variables and community composition/functional profiles were calculated, and correlation heatmaps were visualized using the pheatmap package in R. Differences in soil physicochemical properties and α-diversity were assessed in SPSS using Tukey's HSD test. PCoA based on Bray-Curtis dissimilarities was conducted to evaluate clustering patterns of rhizosphere microbial communities.

## 3 Results

### 3.1 Soil physicochemical properties

Significant differences in soil physicochemical properties were observed among samples (one-way ANOVA followed by Tukey's HSD test, *P* < 0.05). Values are presented as mean ± SD. A clear gradient was detected for salinity-related indicators. The lowest water-soluble total salt and electrical conductivity were recorded in S1 (0.87 ± 0.12 g kg^−1^; 0.47 ± 0.06 mS cm^−1^), which were significantly lower than those in the other samples. Values increased significantly in S2 (40.20 ± 3.73 g kg^−1^; 12.55 ± 1.17 mS cm^−1^) and increased further in S3 and S4. The highest water-soluble total salt was observed in S4 (92.21 ± 4.44 g kg^−1^), whereas electrical conductivity did not differ significantly between S3 and S4 (26.70 ± 3.33 vs. 29.70 ± 1.43 mS cm^−1^). Soil pH ranged from 6.83 to 7.54, and pH values were significantly higher in S2 and S4 than in S1 and S3 (*P* < 0.05). For nutrient-related variables, soil organic matter, total nitrogen, and total phosphorus were highest in S3 (5.32 ± 0.27%, 2.27 ± 0.16 g kg^−1^, and 0.41 ± 0.01 g kg^−1^, respectively), whereas the lowest values were observed in S1. Total potassium remained high and did not differ significantly among S2–S4 (11.34–13.12 g kg^−1^), but was significantly lower in S1 (2.88 ± 0.06 g kg^−1^). According to a China national standard for the classification and grading of soils for agricultural utilization of saline–alkali land (National Standard of the People's Republic of China, 2020), all sites except S1 were classified as severely to extremely severely salinized; accordingly, soils in this study were categorized as low-salinity (S1), moderate-salinity (S2), high-salinity (S3), and very-high-salinity (S4) groups based on salinity levels. The physicochemical properties of the soils are shown in Table 2.

**Table 2:** Physicochemical properties of soil at various sites.

**Name**	**Soil organic matter (%)**	**Total N (g kg^−1^)**	**Total P (g kg^−1^)**	**Total K (g kg^−1^)**	**Total dissolved salts (g kg^−1^)**	**pH**	**EC (mS cm^−1^)**
S1	0.87 ± 0.07 d	0.13 ± 0.01 d	0.18 ± 0.01 d	2.88 ± 0.06 b	0.87 ± 0.12 d	6.83 ± 0.12 b	0.47 ± 0.06 c
S2	2.20 ± 0.09 b	0.08 ± 0.07 b	0.33 ± 0.02 b	11.34 ± 0.46 a	40.20 ± 3.73 c	7.54 ± 0.10 a	12.55 ± 1.17 b
S3	5.32 ± 0.27 a	2.27 ± 0.16 a	0.41 ± 0.01 a	11.76 ± 1.75 a	81.00 ± 10.10 b	7.09 ± 0.30 b	26.70 ± 3.33 a
S4	1.39 ± 0.10 c	0.06 ± 0.02 c	0.22 ± 0.02 c	13.12 ± 0.29 a	92.21 ± 4.44 a	7.42 ± 0.09 a	29.70 ± 1.43 a

Different lowercase letters indicate significant differences (P < 0.05).

### 3.2 Overview of sequencing data

In total, 162.8 Gb of raw metagenomic sequencing data were generated from 12 samples, and, after quality filtering, 161.13 Gb of high-quality data were retained, with an average of 13.43 Gb per sample. After filtering, Q20 values for all samples exceeded 97%, Q30 values exceeded 93%, and the mean GC content was 61.8%, indicating high overall data quality and low sequencing error rates. These data were therefore considered suitable for subsequent high-resolution analyses (Supplementary Table S1).

A total of 9,432,427 non-redundant genes were identified, with a combined length of 5,224.35 Mbp, an average length of 553.87 bp, and an overall GC content of 63.02%. Approximately 77.19% of the total genes were annotated in the NR database, of which 8.65% were unclassified. Among the annotated sequences, archaea accounted for 27.85%, bacteria for 48.80%, eukaryotes for 0.05%, and viruses for 0.49%. At the phylum level, the most abundant groups were Euryarchaeota (31.62% of annotated genes), Proteobacteria (24.18%), Actinobacteriota (5.92%), Bacteroidota (5.88%), Bacillota (2.90%), Pseudomonadota (2.60%), Acidobacteriota (2.12%), Chloroflexota (1.65%), Myxococcota (1.49%), and Planctomycetota (1.08%). At the genus level, the most dominant taxa were *Natronomonas* (2.30%), *Halomicrobium* (1.82%), *Halorussus* (1.63%), *Natrinema* (1.43%), *Fodinibius* (1.29%), *Salinarchaeum* (0.95%), *Halosimplex* (0.93%), *Halobacterium* (0.84%), *Haloterrigena* (0.78%), and *Halomonas* (0.75%).

### 3.3 Composition and structural characteristics of microbial communities

**Figure 1:**
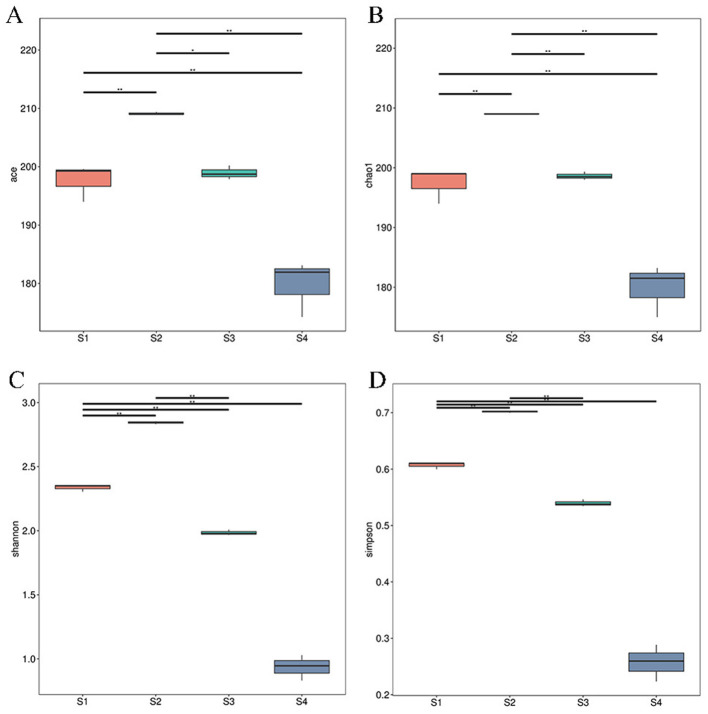
Boxplots of ACE **(A)**, Chao1 **(B)**, Shannon **(C)**, and Simpson **(D)** diversity indices at the phylum level for each sampling site. Asterisks indicate significant differences (*P < 0.05; **P < 0.01; Tukey's test).

To obtain an in-depth assessment of the diversity of *Tamarix* rhizosphere soil microbial communities, alpha-diversity indices, including ACE, Chao1 (richness), Shannon (diversity) and Simpson, were calculated based on the metagenomic sequencing data. The results showed that the mean Shannon index was 1.41 at the phylum level but increased to 4.56 at the genus level, indicating that community structural complexity was higher at finer taxonomic resolution. Similarly, the mean Simpson index was 0.53 at the phylum level and 0.96 at the genus level. Among the four soil salinity levels, samples from S2 exhibited the highest Chao1 and Shannon indices, whereas those from S4 showed the lowest values (Figure 1).

**Figure 2:**
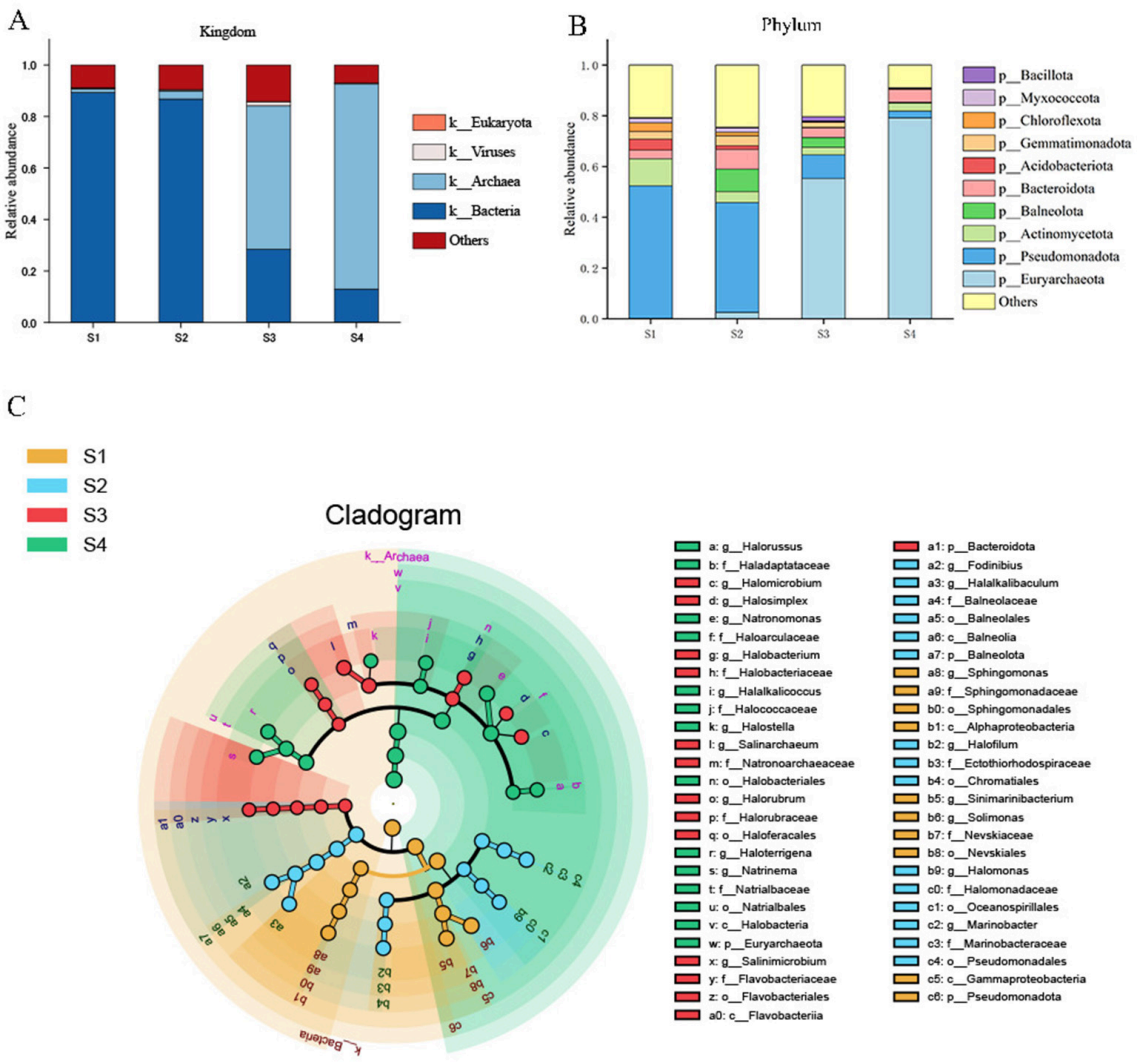
Relative abundances of microbial community composition at the kingdom **(A)** and phylum **(B)** levels in the soil samples, and LEfSe-based cladogram of bacterial taxa **(C)**. Concentric circles from the center to the periphery represent taxonomic levels from kingdom to species.

Taxonomic annotation revealed (Figures 2A, B) that bacterial taxa predominated in low- and medium-salinity soils (S1 and S2), whereas archaeal taxa became dominant in high- and extremely high-salinity soils (S3 and S4). Across all samples, the ten most abundant phyla were Euryarchaeota, *Pseudomonadota*, Actinomycetota, Balneolota, Bacteroidota, Acidobacteriota, Gemmatimonadota, Chloroflexota, Myxococcota, and Bacillota. At the genus level, the most abundant genera were *Natronomonas, Haloterrigena, Solimonas, Halalkalicoccus, Salinarchaeum, Halorussus, Sinimarinibacterium, Natrinema, Halomicrobium*, and *Halostella*.

Based on genus-level relative abundances, the 20 most abundant genera were subjected to linear discriminant analysis effect size (LEfSe) to identify discriminative taxa among the *Tamarix* rhizosphere soil samples (Figure 2C). The results showed that taxa significantly enriched in low-salinity soils (S1) were mainly affiliated with the phylum Pseudomonadota, whereas those enriched in medium-salinity soils (S2) were primarily associated with the phylum Chloroflexota. In high- and extremely high-salinity soils (S3 and S4), taxa belonging to the phylum Bacteroidota together with archaeal lineages became dominant, and in S4 the most strongly enriched taxa were members of the class Halobacteria within the phylum Euryarchaeota.

**Figure 3:**
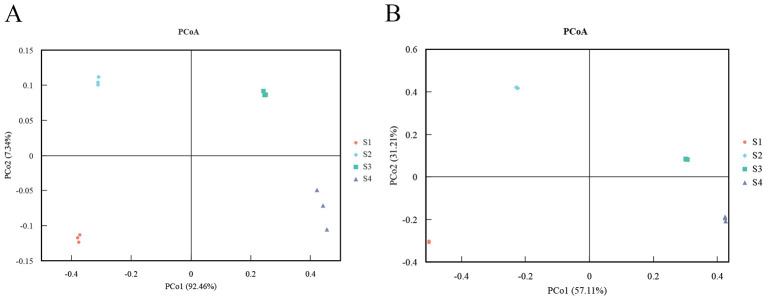
Principal coordinates analysis (PCoA) plots of microbial community composition at the phylum **(A)** and genus **(B)** levels.

To investigate structural differences in *Tamarix* rhizosphere microbial communities among samples, principal coordinates analysis (PCoA) based on Bray-Curtis dissimilarities was performed using phylum- and genus-level relative abundance data. At the phylum level, the PCoA ordination showed clear separation of the four soil samples along the first two axes, indicating pronounced differences in microbial community composition among sampling sites. At the genus level, samples from S3 and S4 clustered in close proximity, suggesting a certain degree of similarity in community composition at this taxonomic resolution. For each site, the three biological replicates clustered tightly together, implying that within-site community structure was relatively stable and that most variation arose from environmental differences among sites (Figure 3).

### 3.4 Functional gene composition analysis

A total of 3,611,342 genes were annotated against the KEGG database. On average, 16.2% of the annotated genes were assigned to the category “Metabolism,” including 4.56% associated with amino acid metabolism, 4.59% with carbohydrate metabolism, 3.10% with energy metabolism, and 2.85% with the metabolism of cofactors and vitamins.

**Figure 4:**
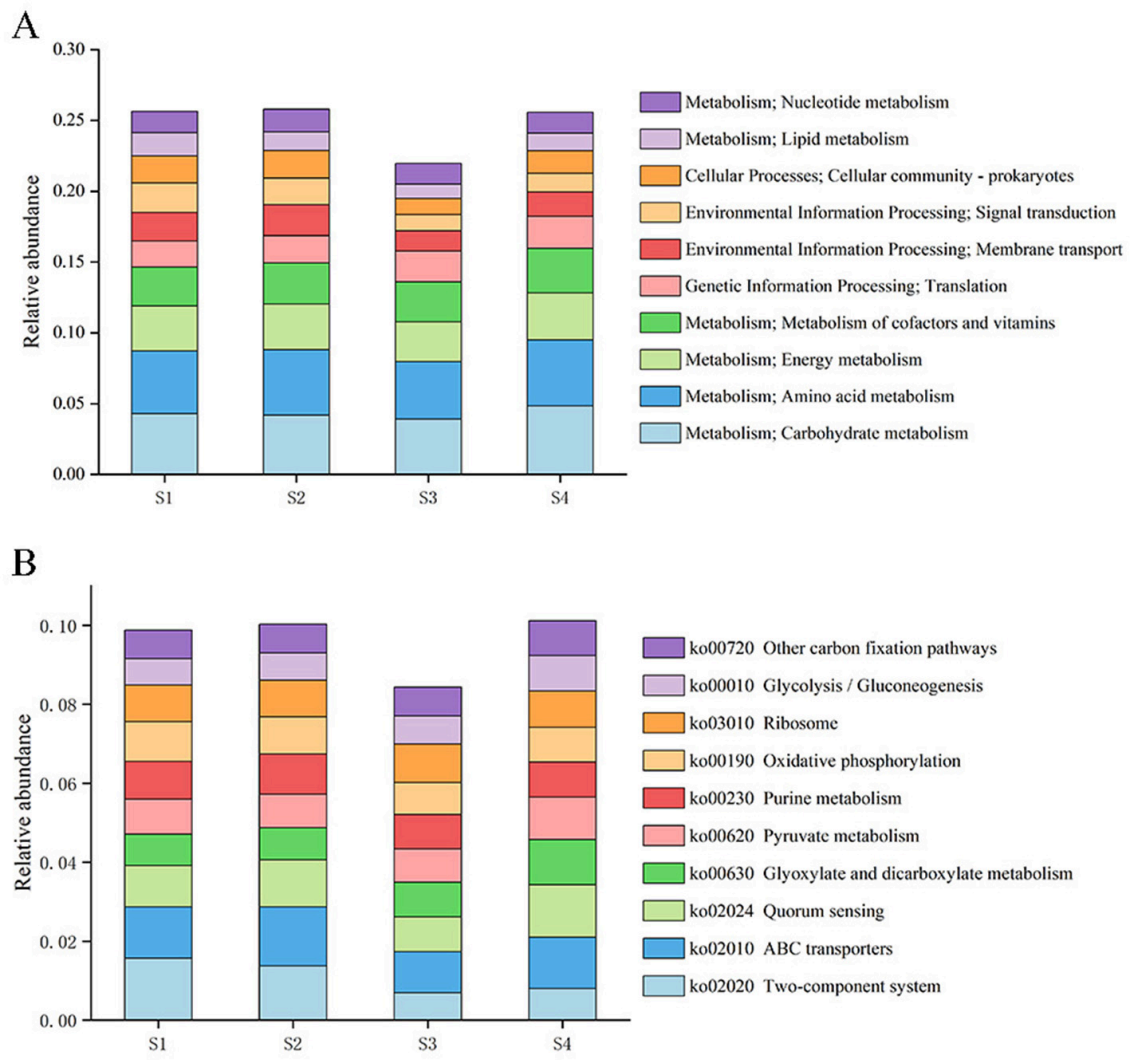
Relative abundances of KEGG functional categories at Level 2 **(A)** and Level 3 **(B)**.

At KEGG Level 1, genes associated with metabolism formed the most abundant category, followed by those involved in genetic information processing, environmental information processing, and cellular processes. As soil salinity increased, the relative abundance of proteins associated with genetic information processing gradually increased, whereas those assigned to organismal systems, environmental information processing, and cellular processes showed a decreasing trend (Figure 4). At KEGG Level 2 (Figure 4A), the most abundant functional categories were proteins involved in carbohydrate metabolism and amino acid metabolism, followed by those related to energy metabolism and the metabolism of cofactors and vitamins. Within the genetic information processing category, genes involved in transcription, translation, replication, and repair increased in relative abundance with increasing salinity, whereas several subcategories related to environmental information processing (membrane transport and signal transduction), metabolism (lipid metabolism, glycan biosynthesis and metabolism, and other amino acid metabolism), and cellular processes (cell motility and cell growth and death) decreased (Supplementary Table S2).

A total of 441 KEGG Level 3 pathways were annotated (Figure 4B), and the most enriched pathways were associated with ABC transporters, two-component systems, quorum sensing, pyruvate metabolism, purine metabolism, and glyoxylate and dicarboxylate metabolism. Genes involved in two-component systems, secretion systems, and peptidoglycan biosynthesis showed a decreasing trend with increasing salinity, whereas pathways related to pyruvate metabolism, methane metabolism, carbon fixation, glycolysis/gluconeogenesis, and glyoxylate and dicarboxylate metabolism exhibited an increasing trend (Supplementary Table S3).

**Figure 5:**
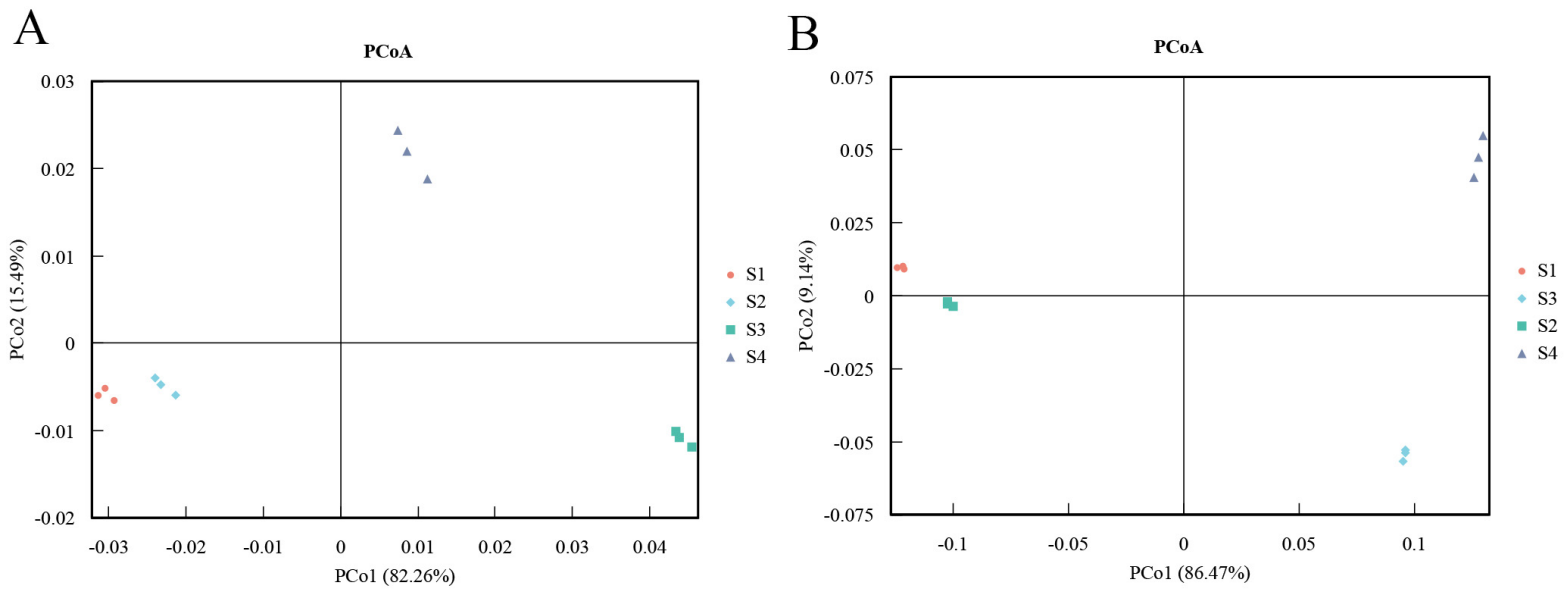
Principal coordinates analysis (PCoA) plots based on KEGG Level 2 functional categories **(A)** and KEGG ortholog (KO) level **(B)**.

Principal coordinates analysis (PCoA) based on Bray-Curtis dissimilarities was performed using gene abundance profiles at the KEGG Level 2 and KEGG orthology (KO) levels. As shown in Figure 5, samples from S1 and S2 clustered in close proximity in the PCoA ordination, indicating that the functional gene composition at these sites was similar and that their microbial communities exhibited a high degree of similarity in metabolic functions. In contrast, samples from S3 and S4 were more widely separated in the PCoA plot, suggesting greater differences in functional profiles between these two sites.

**Figure 6:**
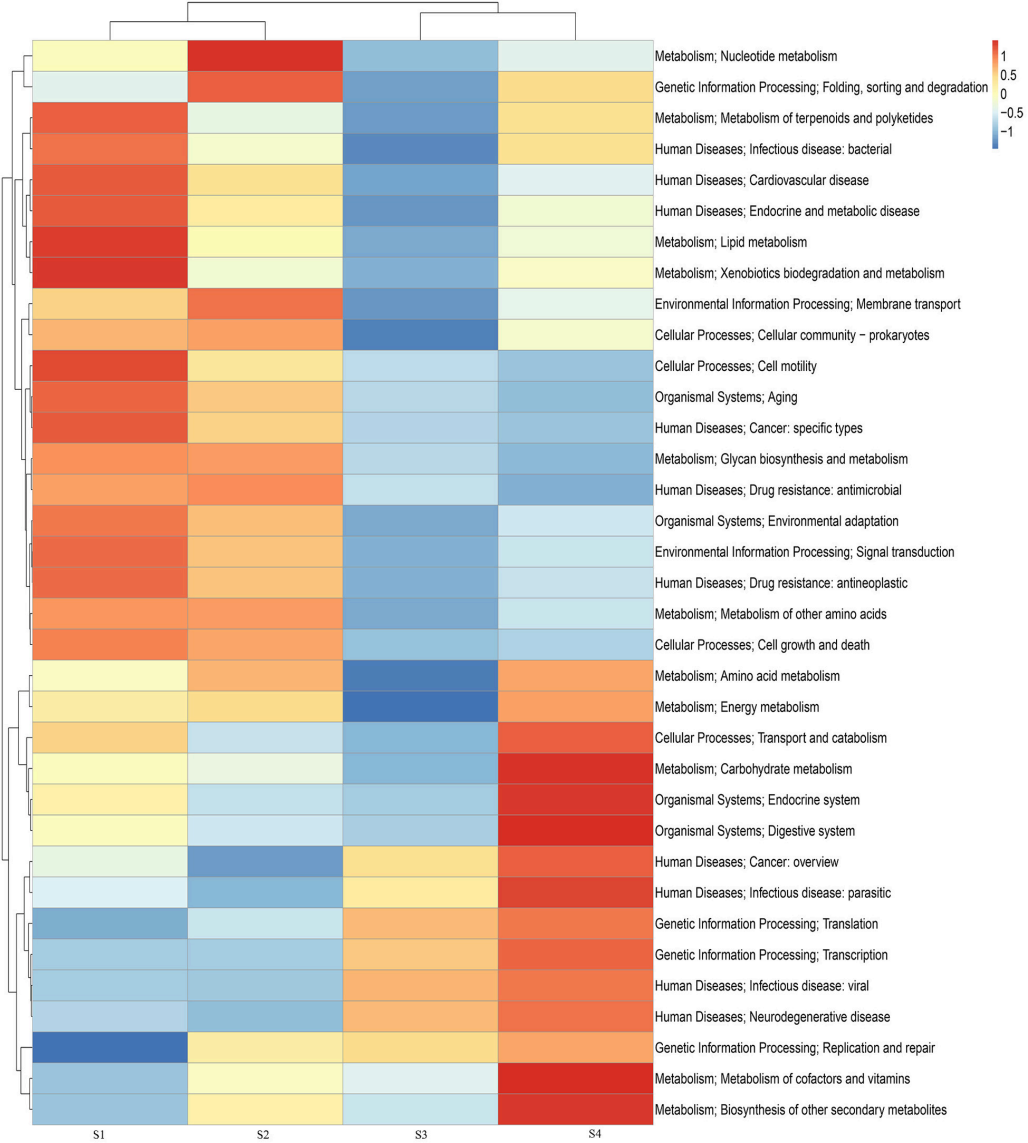
Heatmap of KEGG Level 2 functional pathways for the different samples (DESeq2, Benjamini–Hochberg adjusted FDR < 0.05). Heatmap values are row-wise *Z*-scores of pathway relative abundances, and the left dendrogram represents hierarchical clustering based on functional profiles.

As shown in the heatmap (Figure 6), at KEGG Level 2, genes associated with the metabolism of cofactors and vitamins, transcription, translation, carbohydrate metabolism, and the biosynthesis of other secondary metabolites were more abundant in higher-salinity soils than in low-salinity soils, whereas genes involved in nucleotide metabolism, lipid metabolism, cell motility, and glycan biosynthesis and metabolism were more abundant in low-salinity soils than in extremely high-salinity soils. Genes involved in translation, transcription, replication, and repair increased in relative abundance with increasing salinity, whereas genes associated with glycan biosynthesis and metabolism showed a decreasing trend. At the KEGG Level 3 pathway level, low-salinity soils showed significantly higher relative abundances of pathways related to bacterial two-component systems, benzoate degradation, peptidoglycan biosynthesis, bacterial secretion systems, and homologous recombination than high-salinity soils, whereas extremely high-salinity soils were enriched in pathways such as methane metabolism, pyruvate metabolism, glyoxylate and dicarboxylate metabolism, the pentose phosphate pathway, glycolysis/gluconeogenesis, carbon fixation pathways, and phenylalanine, tyrosine, and tryptophan biosynthesis.

**Figure 7:**
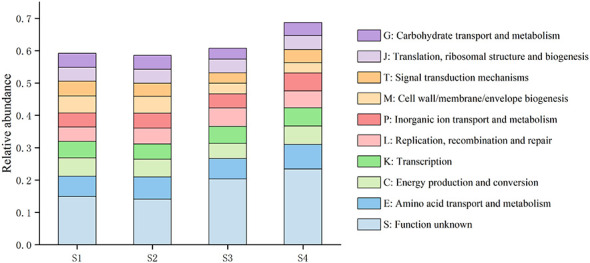
Relative abundances of functional categories annotated at eggNOG Level 1.

In terms of orthologous group annotation, 79.4% of the predicted genes were annotated against the eggNOG database, among which 10.83% were assigned to amino acid transport and metabolism, 8.38% to energy production and conversion, 8.23% to replication, recombination, and repair, 7.10% to transcription, 7.01% to cell wall/membrane/envelope biogenesis, and 6.76% to carbohydrate transport and metabolism (Figure 7). With increasing soil salinity, proteins associated with replication, recombination, and repair, amino acid transport and metabolism, translation, transcription, and ribosomal structure and biogenesis increased in relative abundance, whereas those involved in intracellular trafficking, secretion, and vesicular transport decreased (Figure 7).

**Figure 8:**
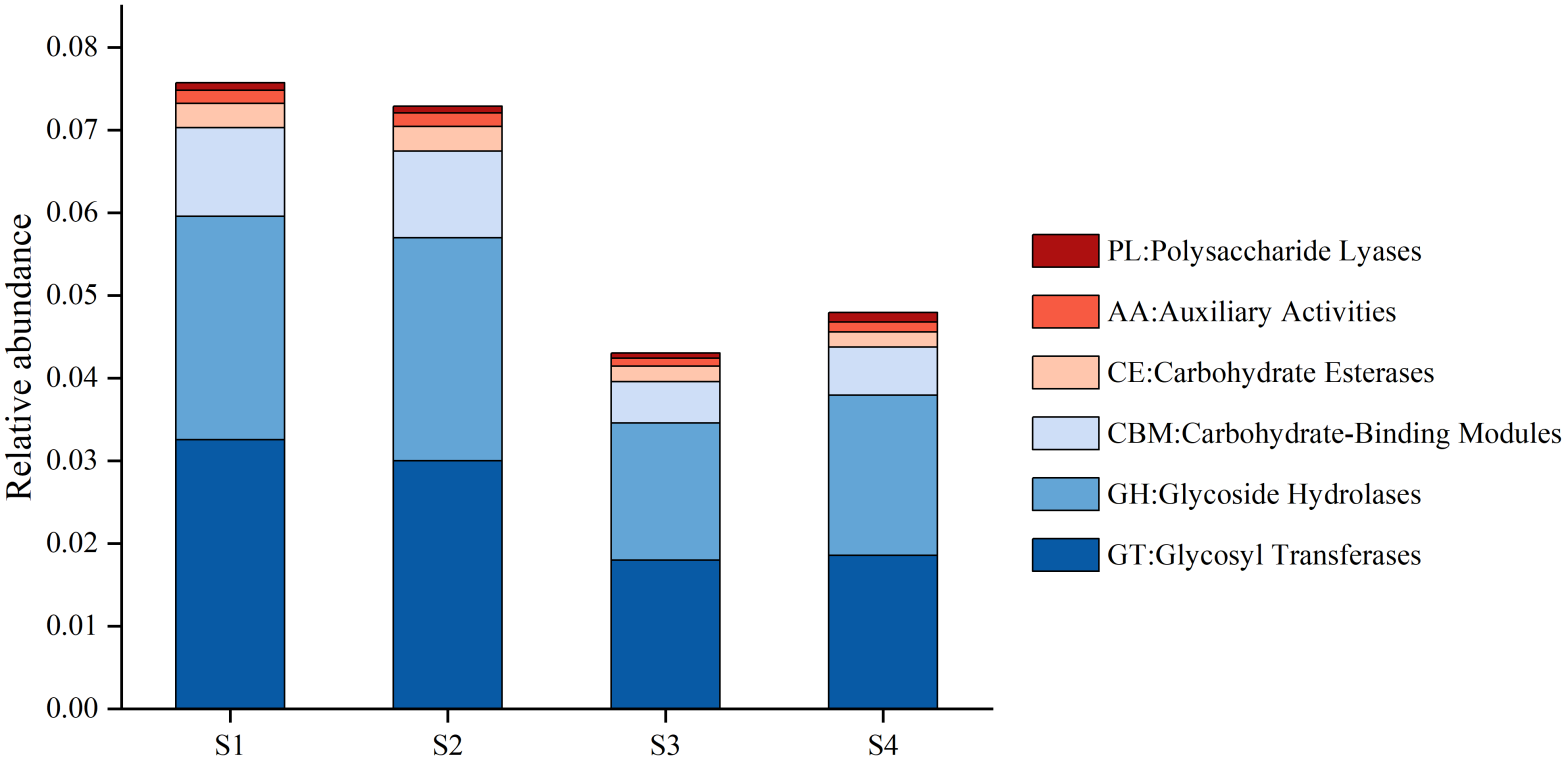
Relative abundances of functional categories annotated at CAZy Level 1.

In terms of carbohydrate-active enzyme annotation, a total of 580,387 genes were assigned to CAZy families, with glycosyltransferases (GTs), glycoside hydrolases (GHs), and carbohydrate-binding modules (CBMs) being the most abundant classes (Figure 8). By contrast, carbohydrate esterases (CEs), polysaccharide lyases (PLs), and auxiliary activities (AAs) were present at relatively low abundances. In extremely high-salinity soils, the relative abundance of PLs increased, whereas all other CAZy families showed lower abundances than in low- and medium-salinity soils, suggesting that increasing soil salinity may reduce the overall potential for carbohydrate-active enzyme functions in the *Tamarix* rhizosphere.

### 3.5 Association analysis of microbial community structure and environmental variables

**Figure 9:**
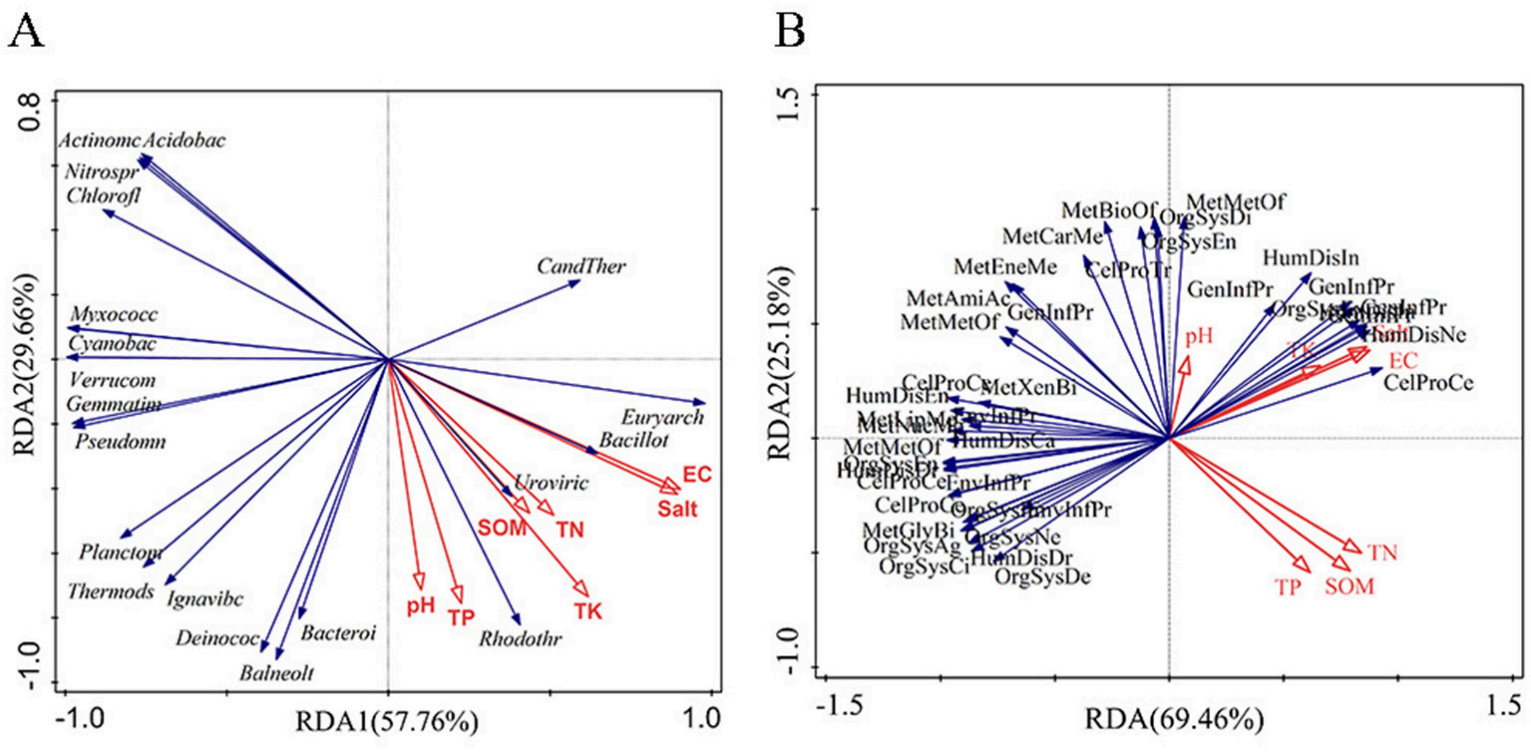
Redundancy analysis (RDA) of the relationships between soil physicochemical factors and microbial community structure at the phylum level **(A)** and KEGG Level 2 functional profiles **(B)**.

The RDA results indicated that differences in community structure among samples were significantly explained by soil physicochemical variables. Electrical conductivity (EC) accounted for the largest proportion of explained variation (52.1%, *P* = 0.002) and was identified as the primary driver, whereas total potassium (TK; 32.3%, *P* = 0.004) and soil organic matter (SOM; 13.5%, *P* = 0.002) also contributed significantly. Total salt (Salt) explained a smaller yet significant fraction of variation (1.1%, *P* = 0.002), and total phosphorus (TP) showed a modest but significant contribution (0.4%, *P* = 0.028). In contrast, total nitrogen (TN) and pH were not significant (*P* > 0.05). Overall, rhizosphere community differentiation was dominated by a salinity-related gradient, as represented by EC (Figure 9A). At KEGG Level 2, the RDA analysis showed that EC remained the strongest explanatory variable (57.3%, *P* = 0.002), followed by SOM (31.5%, *P* = 0.002). TK provided an additional independent contribution (9.3%, *P* = 0.004), and TP explained a small but significant fraction (0.7%, *P* = 0.006). By comparison, Salt, TN, and pH did not reach statistical significance in this model (*P* > 0.05). Together, these results suggest that functional-profile differentiation was jointly driven by a salinity-related gradient and resource availability (SOM; Figure 9B) (Supplementary Table S6).

**Figure 10:**
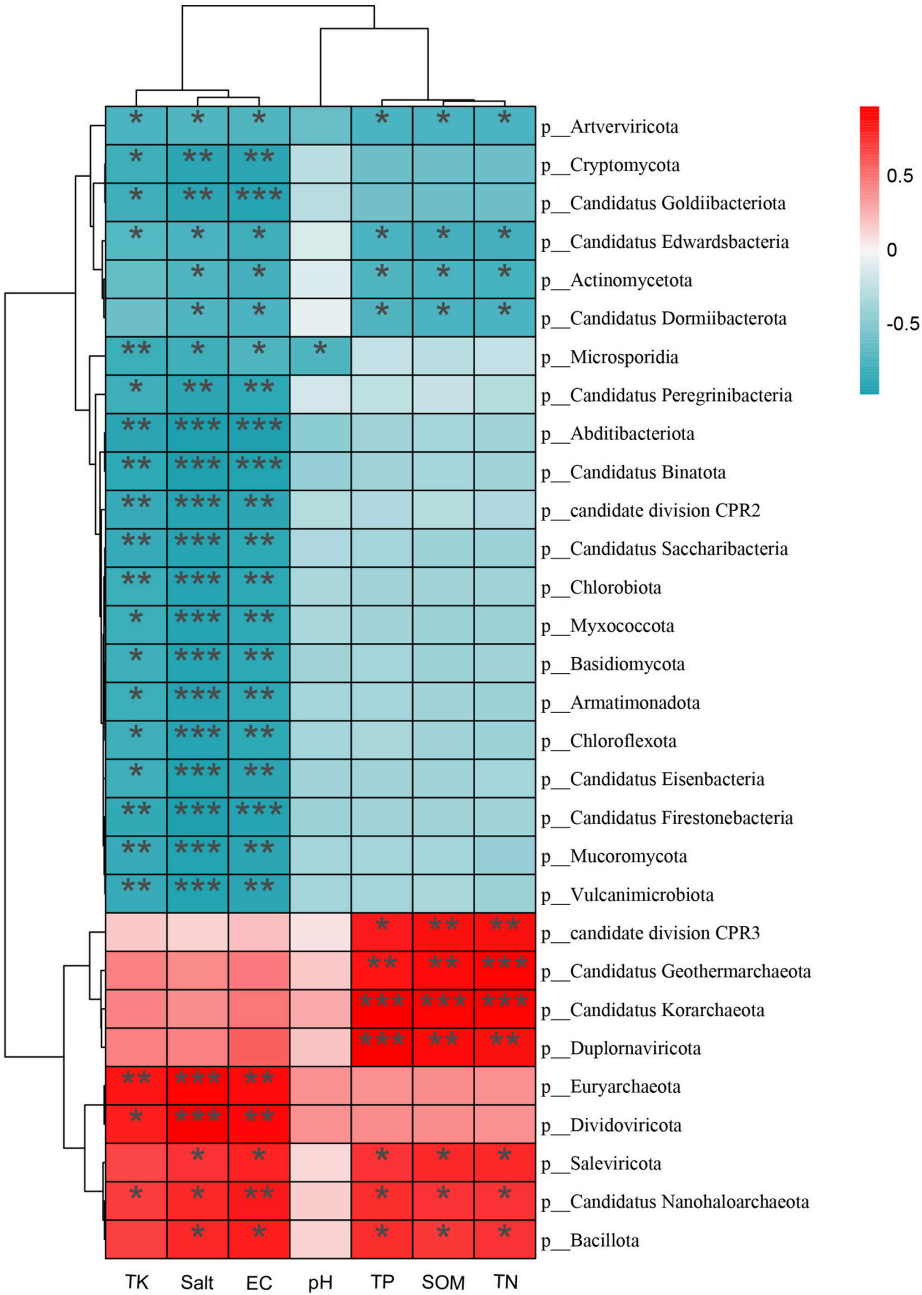
Heatmap of correlations between soil physicochemical factors and microbial community composition at the phylum level (*P < 0.05, **P < 0.01, ***P < 0.001).

To evaluate associations between soil physicochemical variables and community composition, the top 30 most abundant phyla were selected, and Spearman's rank correlations were calculated with FDR adjustment (Figure 10). Clustering of environmental variables showed that salinity-related indicators (Salt and EC) grouped with certain ionic/mineral factors (e.g., TK), representing a major salinity/ionic-strength gradient, whereas nutrient-related variables (TP, SOM, and TN) formed a relatively independent branch. Overall, most bacterial phyla were negatively correlated with Salt/EC (*P* < 0.05), indicating that their relative abundances tended to decrease with increasing salinity. In contrast, several archaeal and halotolerance-associated lineages (e.g., Halobacteriota, Euryarchaeota, and Nanoarchaeota) were positively correlated with Salt/EC (*P* < 0.05), suggesting that they were selectively enriched under high-salinity rhizosphere conditions. Beyond salinity, SOM, TN, and TP were also significantly associated with several phyla (*P* < 0.05), indicating that nutrient status may exert a synergistic influence on community distribution under salinity-driven filtering (Supplementary Table S4).

**Figure 11:**
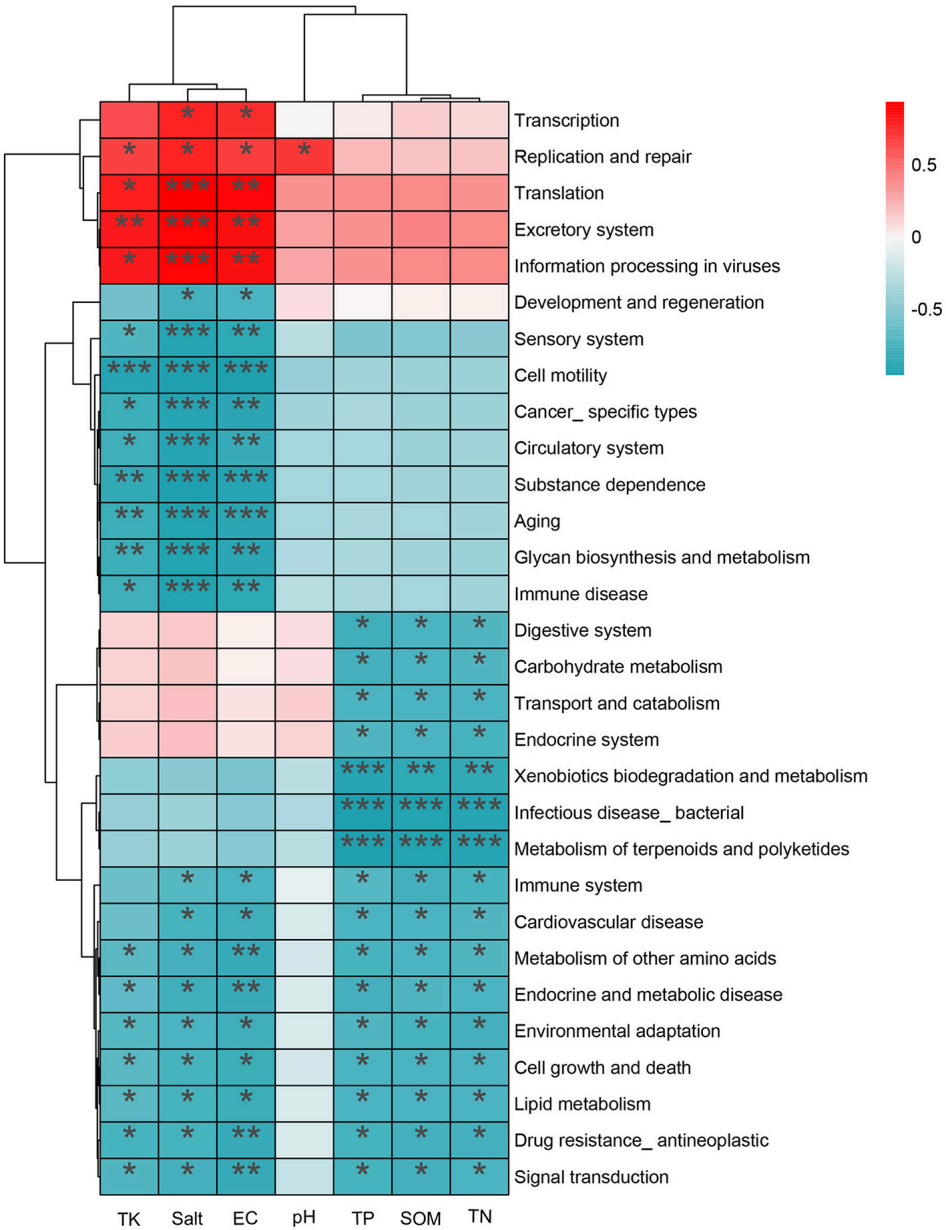
Heatmap of correlations between soil physicochemical factors and KEGG Level 2 functional categories (*P < 0.05, **P < 0.01, ***P < 0.001).

Similarly, significant association patterns were observed between physicochemical variables and KEGG Level 2 functional profiles (Figure 11). Salt/EC were positively correlated with genetic information processing functions (Transcription, Translation, and Replication and repair; *P* < 0.05), indicating a shift toward functional potential related to maintenance and repair under stronger salinity stress. By contrast, Lipid metabolism, Signal transduction, Cell growth and death, and Xenobiotics biodegradation and metabolism were negatively correlated with salinity indicators (*P* < 0.05), suggesting that functional allocation shifted from growth/metabolic expansion toward stress adaptation and homeostasis. In addition, TP, SOM, and TN were significantly associated with several metabolism-related functional categories (*P* < 0.05), indicating that functional composition was influenced not only by salinity gradients but also by resource availability (Supplementary Table S5).

### 3.6 Salt tolerance gene analysis

**Figure 12:**
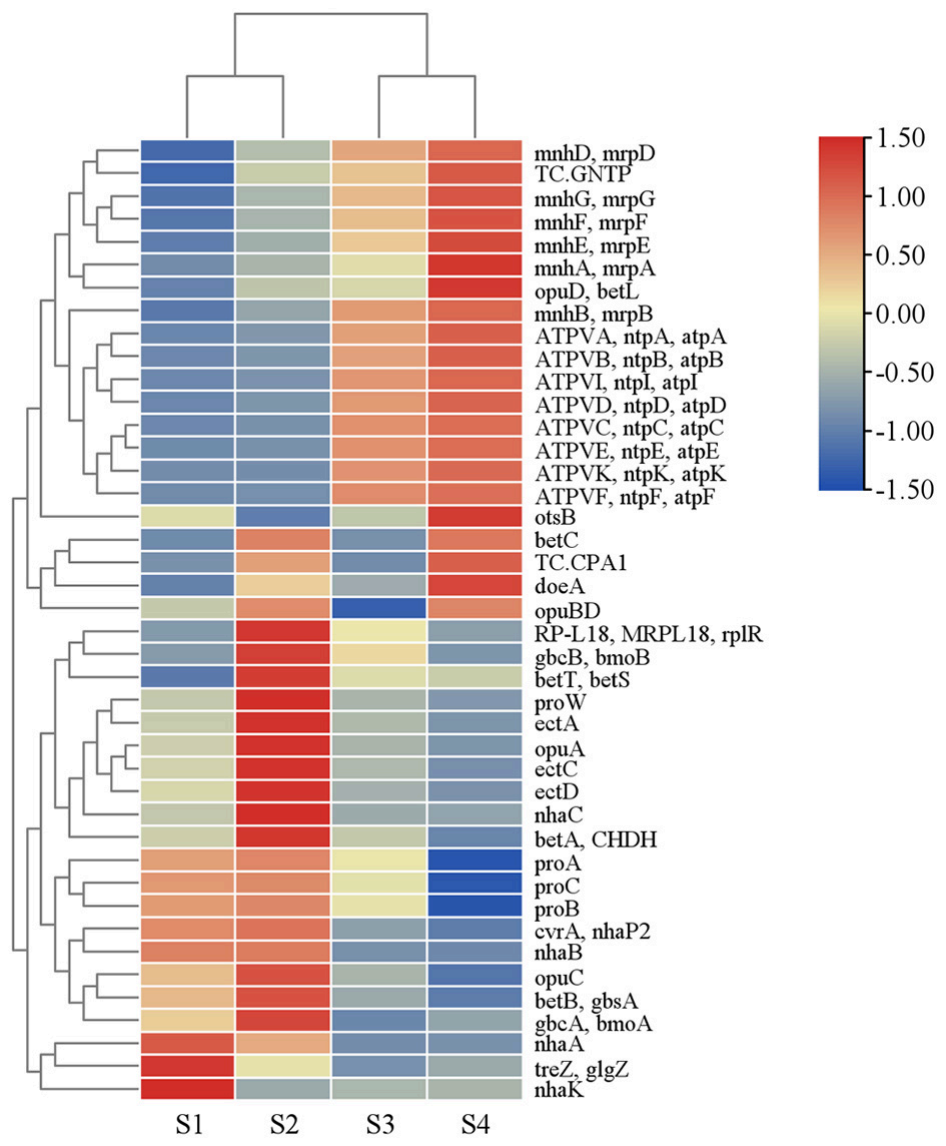
Heatmap of salt stress-related genes with row-wise normalization.

In this study, a subset of salt stress–related genes was identified in the four *Tamarix* rhizosphere soil samples, including Na^+^/H^+^ antiport systems (*nhaA, nhaB, nhaK*, and the *mnh/mrp* multi-subunit complexes), genes involved in betaine synthesis and transport (*betA, betB, betC, gbcA, gbcB, opuA, opuB, opuC, opuD, betT*, and *betL*), proline biosynthesis genes (*proA, proB*, and *proC*), and ectoine synthesis genes (*ectA, ectB, ectC*, and *ectD*), indicating that the rhizosphere microbial community possesses a multilayered adaptive potential to salt stress (Supplementary Table S7). Across the four soil salinity levels, the total abundance of salt tolerance–related genes increased significantly with increasing salinity (Figure 12).

At the level of specific gene functions, Na^+^/H^+^ antiport systems were markedly enriched in the S3 and S4 groups, particularly the *mnh/mrp* multi-subunit Na^+^ pump complexes, indicating that in extremely saline environments microbes rely on enhanced Na^+^ efflux and maintenance of the proton gradient to achieve ionic homeostasis. At the same time, genes involved in betaine synthesis and transport were significantly enriched in the S2 group, and the proline biosynthesis genes *proA, proB* and *proC* showed a similar pattern, suggesting that compatible solute accumulation plays a key role in salt tolerance of the *Tamarix* rhizosphere microbiome. In addition, ectoine synthesis genes decreased markedly in the S3–S4 groups (Roberts, 2005; Czech et al., 2018), representing a typical high-salinity response feature. Meanwhile, several V/A-type ATPase genes increased in abundance under high-salinity conditions, implying that microbes increasingly depend on energy-dependent proton pumps to maintain membrane potential and osmotic balance between the intracellular and extracellular environments.

## 4 Discussion

### 4.1 Changes in rhizosphere microbial community structure

At the species level, the *Tamarix* rhizosphere microbiome exhibited a generally high baseline diversity, but a declining trend was observed with increasing salinity, suggesting that salinity stress exerted strong filtering and inhibitory effects on community structure. Alpha-diversity metrics showed that Shannon indices exceeded 5.0 across all four sites, and Simpson indices were generally >0.97, indicating high species-level diversity. Meanwhile, diversity and community richness decreased overall along the salinity gradient, consistent with the notion that salt accumulation can restrict the growth and establishment of salt-sensitive taxa via osmotic stress and ion toxicity, thereby promoting a shift from a relatively diverse community toward a more halotolerant structure (Zhang et al., 2019; Chen et al., 2022). This pattern is consistent with previous reports (Liu et al., 2025). This interpretation was further supported by PCoA and RDA, in which community structures were significantly separated among sites (*P* < 0.01). Moreover, salinity-related gradients (e.g., EC and total salt) were closely associated with community dissimilarity, indicating that salinity stress was a major environmental factor shaping rhizosphere community variation.

The very-high-salinity treatment showed the lowest number of detected species (14,915), further suggesting that high salinity constrained microbial colonization and proliferation and may have reduced the community's “maintainable richness” by lowering substrate-use efficiency. Previous studies have reported that high-salinity conditions are often associated with reduced microbial carbon-use efficiency and lower extracellular enzyme activities (Dong et al., 2022), which can limit organic matter decomposition and carbon-metabolic efficiency and thereby suppress microbial biomass formation (Ning et al., 2024). In addition, increasing salinity may alter both the composition and supply of root exudates (e.g., soluble sugars, organic acids, and amino acids) (Kumar et al., 2023), thereby reshaping the availability of rhizosphere substrates and the formation of nutrient “hotspots,” and ultimately influencing microbial colonization and community turnover (Yang et al., 2025). Consistently, studies in saline–alkaline soils have shown that high salinity imposes substantial constraints on microbial colonization and growth (Wang et al., 2020).

Along the salinity gradient, the relative abundances of bacterial and archaeal taxa changed in opposite directions, suggesting differences in salinity tolerance and niche occupancy between the two domains. In low-salinity soils, the relative abundances of Pseudomonadota, Actinobacteriota, and Bacteroidota were 41.63, 12.67, and 4.83%, respectively, and these phyla remained dominant across the full sample set. Previous studies have reported that certain members of Pseudomonadota (Ghadamgahi et al., 2022; Saha and Sen, 2024), Actinobacteriota (Palaniyandi et al., 2014; Srivastava et al., 2014), and Bacteroidota (Lidbury et al., 2020) exhibit salinity tolerance and organic-matter degradation potential, and may indirectly support plant growth by enhancing organic matter turnover, increasing nutrient availability, and/or exerting plant growth–promoting effects. Accordingly, the relative enrichment of these dominant phyla under low-salinity conditions may reflect a rhizosphere in which material turnover and plant-microbe interactions remain comparatively active; however, this interpretation should be viewed as inferential rather than definitive.

Under high- and very-high-salinity treatments, the relative abundance of Euryarchaeota increased significantly, suggesting that archaea may be more tolerant of intense salinity stress and could contribute to key ecological processes under such conditions. Archaea are known to play important roles in rhizosphere and soil C and N cycling; for example, ammonia-oxidizing archaea participate in nitrogen transformation processes (Schleper and Nicol, 2010; Wright and Lehtovirta-Morley, 2023), and methanogenic archaea contribute substantially to carbon cycling (Evans et al., 2019). Therefore, the salinity-associated increase in archaea may help sustain certain baseline ecosystem functions under stress and may enhance rhizosphere stability following community reassembly, potentially through resource competition and niche occupancy rather than through unverified suppression of specific harmful microbes.

While salinity acted as the dominant environmental filter, the *Tamarix* rhizosphere itself may have further steered community assembly via a “rhizosphere effect,” thereby jointly shaping community differences across salinity levels (Berendsen et al., 2012). The rhizosphere, as a highly active microdomain at the plant-soil interface, is typically characterized by steeper chemical gradients and stronger resource inputs. First, ion uptake and exchange by roots can modify rhizosphere pH and ionic composition (including local distributions of salts and trace elements), thereby altering microbial niche conditions and influencing the colonization of different taxa (Wang et al., 2023). Second, root exudates (e.g., soluble sugars, organic acids, amino acids, and diverse secondary metabolites) supply labile carbon and create nutrient “hotspots,” which can selectively enrich chemotactic microorganisms that efficiently utilize rhizosphere carbon or possess stress-tolerance traits, thereby reshaping dominant lineages (Bais et al., 2006). In addition, the *Tamarix* rhizosphere may regulate the bioavailability of trace elements (e.g., Fe) through the release of metal-chelating compounds/ion carriers (such as siderophores) and other specific chemicals, thereby modulating microbial interactions and potentially influencing community stability (Saha et al., 2015). Taken together with our environmental-factor results, a salinity gradient appeared to provide the dominant filtering, whereas rhizosphere processes may further influence the direction of community colonization and turnover through microenvironmental modification and exudate inputs (Hu et al., 2018).

### 4.2 Changes in microbial functional composition under salt stress

Under salinity stress, the functional profile of the *Tamarix* rhizosphere microbiome remained dominated by metabolism-related functions, suggesting that substantial potential for material transformation was retained and that a degree of metabolic stability was maintained. KEGG-based functional annotation showed that metabolism-related genes accounted for a large proportion across all samples, consistent with findings from many soil microbiome studies (Feng et al., 2018). This pattern suggests relatively active transformation and metabolic capacity in the rhizosphere community (Xiao et al., 2021). Despite pronounced differences along the salinity gradient, core metabolic categories such as amino acid metabolism and energy metabolism remained highly represented, implying that microorganisms may preferentially maintain basal metabolic networks to support essential cellular functions under combined drought-salinity stress. This observation is consistent with reports from arid ecosystems and saline-alkaline soils indicating relative stability of core metabolism (Dai et al., 2019; Qi et al., 2023).

With increasing salinity stress, functions related to genetic information processing and cellular maintenance/repair showed an increasing trend, suggesting that more resources were allocated to homeostasis under stress. Under high-salinity conditions, genes involved in translation, transcription, and DNA replication and repair were enriched (Capes et al., 2011), consistent with the eggNOG annotation showing increases in protein-repair and stress-response proteins. Together, these patterns suggest that salinity stress may increase protein misfolding, oxidative pressure, and the risk of DNA damage, thereby promoting enhanced protein synthesis and chaperone-mediated folding and repair to maintain cellular function (Hahne et al., 2010; Yang et al., 2022). Similar responses have been observed in other systems. For example, algal cells can adapt to salinity stress by enhancing transcriptional regulation and protein folding (Zhang et al., 2022), and upregulation of proteins involved in protein/nucleic-acid synthesis and repair under salinity stress has also been reported (Naamala et al., 2023).

In parallel with the enhancement of maintenance/repair functions, energy-intensive processes related to environmental sensing, motility, and collective behaviors declined markedly in high-salinity samples, suggesting a shift from a “resource acquisition-growth expansion” strategy toward an “energy conservation-homeostasis maintenance” strategy. Specifically, pathways such as Two-component system, Membrane transport, Flagellar assembly, Biofilm formation, and Peptidoglycan biosynthesis were downregulated under high-salinity treatments (Philips et al., 2017). This pattern suggests that chemotaxis/motility, biofilm construction, and cell-wall biosynthesis may be reduced to lower energetic costs, allowing limited resources to be preferentially allocated to osmotic balance and stress defense, thereby potentially increasing survival under prolonged salinity stress (Guo et al., 2022; Feng et al., 2022; Fu et al., 2014). This trade-off has also been supported by experimental evidence. For example, high-salinity exposure has been shown to impair bacterial aggregation and biofilm formation (Novello et al., 2022). In halotolerant bacteria, downregulation of flagellar structures and certain metabolism-related proteins, together with upregulation of stress-defense proteins under salinity stress, has been reported, suggesting that motility can be “abandoned” to conserve energy while reinforcing defensive maintenance.

Pathway-level shifts further suggest that under high salinity, biosynthetic investment was reduced and the carbon-metabolic network was compensatorily reconfigured. In high-salinity samples, pathways related to lipid metabolism, nucleotide metabolism, and glycan biosynthesis were decreased, implying that energy was reallocated from rapid growth and biosynthesis toward cellular homeostasis and stress responses. Meanwhile, glyoxylate and dicarboxylate metabolism (ko00630) and glycolysis/gluconeogenesis (ko00010) were enriched under high salinity (Arense et al., 2010), suggesting that when carbon sources are limited or energy demand increases, metabolism may be sustained by enhanced utilization of non-carbohydrate substrates and reinforced energy-supplying routes (Danevčič and Stopar, 2011; Guo et al., 2023; Zhang et al., 2021). Consistent with these observations, previous work has shown that salinity stress can be accommodated through regulation of genes in key pathways, including carbon metabolism (ko01200), the TCA cycle (ko00020), the pentose phosphate pathway (ko00030), glycolysis/gluconeogenesis (ko00010), and arginine and proline metabolism (ko00330) (Pinner et al., 1993).

The increasing proportion of the “Function unknown” category with stronger salinity stress suggests that the halophytic *Tamarix* rhizosphere may harbor an under-annotated reservoir of functional genes, providing clues for subsequent exploration of salinity-tolerance mechanisms. This increase likely reflects, first, that current reference databases provide limited coverage of functional diversity in halophytic rhizosphere microbiomes and, second, that salinity-selected, lineage- or habitat-specific tolerance-related genes or regulatory modules may remain insufficiently characterized. Consequently, these unassigned functions highlight potential targets for future discovery of salinity-adaptive microbial resources and functional genes.

### 4.3 Effects of environmental factors on community structure and function

RDA results showed that salinity-related indicators, represented by electrical conductivity (EC), explained the largest and statistically significant proportion of variation in both community structure and functional composition, indicating that salinity was the dominant driver of community differentiation and functional reconfiguration. This finding was consistent with the observed decline in α-diversity and the shift from bacterial dominance toward halotolerant archaea, suggesting that salinity stress imposed strong ecological filtering via osmotic pressure and ion toxicity (Castelán-Sánchez et al., 2020). Meanwhile, soil nutrients and mineral elements (e.g., SOM and TK) may also contribute to community and functional variation, implying potential synergistic or antagonistic interactions between salinity and nutrient status (Li X. et al., 2024; Hua et al., 2024). In particular, under high salinity, nutrient heterogeneity may alter the supply of available rhizosphere substrates, thereby influencing microbial colonization and metabolic allocation. Therefore, while salinity-driven effects are interpreted, the coupled influence of salinity and nutrient conditions on rhizosphere microbial processes should also be considered.

Overall, differences in rhizosphere microbial community structure and functional composition were significantly explained by soil physicochemical variables, with salinity identified as the primary driver. At the community-structure level, EC accounted for the highest explained variation (Explains = 52.1%, *P* = 0.002), indicating that an ionic-strength/salinity-stress gradient exerted the strongest environmental filtering on community assembly. At the functional level (KEGG Level 2), EC remained the leading explanatory variable (Explains = 57.3%, *P* = 0.002), suggesting that salinity stress not only shapes which taxa become dominant but also drives systematic differentiation of functional profiles along the salinity gradient. These results were consistent with the clear site separation observed in the PCoA/RDA ordinations and the overall pattern of salinity-associated restructuring of community structure and function, supporting salinity stress as the central environmental context underlying rhizosphere microbial variation in this study.

Water-soluble total salt (Salt) was significant but explained only a small fraction of variation in community structure, and was not significant at the functional level, indicating that salinity effects were captured more effectively by EC as an integrative proxy. In the community-structure RDA, the unique contribution of Salt was 1.1% (*P* = 0.002), whereas in the functional RDA (forward selection) its explained variation decreased further and became non-significant (Explains = 0.4%, *P* = 0.162). This pattern may reflect collinearity between EC and Salt. In salinized soils, EC often more sensitively reflects the overall strength of soluble ions, whereas total salt, as a mass-based measure, may retain only a small additional independent contribution in multivariable models. Accordingly, salinity-related gradients, with EC used as a proxy, dominated the differentiation of both community structure and functional composition, and the contribution of total salt was largely absorbed in models that included EC, resulting in a low or non-significant unique effect.

Beyond the salinity gradient, resource and nutrient conditions (SOM, TK, and TP) also exerted significant secondary effects on community structure and functional composition, suggesting that rhizosphere structure-function differentiation was jointly shaped by stress and resource availability. In the community-structure RDA, TK and SOM explained 32.3% (*P* = 0.004) and 13.5% (*P* = 0.002) of the variation, respectively, indicating that beyond salinity-driven filtering, the ionic/nutritional context and organic-substrate supply also influenced niche occupancy of dominant taxa and community turnover. At KEGG Level 2, SOM accounted for 31.5% of the explained variation (*P* = 0.002), TK contributed significantly (9.3%, *P* = 0.004), and TP, although explaining a smaller fraction, remained significant (0.7%, *P* = 0.006). These results suggest that functional-profile differences were driven not only by salinity stress but also by substrate supply and nutrient status. Conceptually, salinity sets the direction and intensity of environmental filtering, whereas SOM and nutrient availability provide the resource context for functional allocation, leading to site-specific differences in metabolic investment, maintenance/repair capacity, and biogeochemical cycling potential.

### 4.4 Mechanisms underlying microbial salt tolerance

Along the high-salinity gradient, halotolerance-associated genes suggested that rhizosphere microorganisms were primarily adapted to extreme salinity stress by strengthening energy-dependent ion-homeostasis systems (Wang Y. et al., 2025). Halotolerance gene profiling showed that V/A-type ATPases and the multicomponent mnh/mrp Na^+^/H^+^ antiporter system increased significantly with increasing soil salinity, indicating that under very high salinity, greater energy investment was required to actively pump H^+^ and/or extrude Na^+^ to maintain intracellular ion balance and membrane potential stability. V/A-type ATPases were inferred to drive transmembrane ion transport using ATP and may participate in energy conversion under specific conditions. The mnh/mrp system, acting as a multicomponent antiporter, was inferred to facilitate the extrusion of excess Na^+^; together, these systems were expected to support cellular homeostasis and enhance salinity tolerance. By contrast, single-gene nha-type antiporters (e.g., nhaA, nhaB, and nhaK) decreased in abundance under very high salinity, suggesting that ion-regulation strategies may be tiered across salinity regimes (Pinner et al., 1993). Specifically, under low salinity, a relatively “simplified” nha-type transport mode may predominate, whereas under very high salinity, reliance may shift toward structurally complex and energy-dependent mnh/mrp and ATPase systems to achieve stronger ion-homeostasis capacity (Arkin et al., 2007).

In contrast to the strengthened ion-homeostasis systems, pathways related to compatible-solute biosynthesis declined overall in high- and very-high-salinity samples, suggesting that osmoprotection strategies may shift from “biosynthesis-based” to “uptake/usage-based” modes. Betaine and ectoine are common osmoprotectants (Talibart et al., 1994); however, genes associated with their biosynthesis were generally reduced under high and very high salinity, indicating that these biosynthetic routes may be downregulated or become less important under stronger salinity stress. Given that compatible-solute synthesis is typically metabolically costly, these results suggest that under high salinity, energy-intensive biosynthetic processes may be curtailed, with a greater reliance on uptake of exogenous compatible solutes or alternative osmotic-regulation mechanisms to maintain osmotic balance. Previous studies have also reported that betaine-related pathways can be more active at moderate salinity but may be inhibited at higher salinity, consistent with the trend observed here (Srivastava et al., 2022).

Differential patterns in proline-related genes further suggest that osmotic-regulation pathways may exhibit a “stage-specific response” across salinity levels rather than a monotonic trend. Genes involved in proline biosynthesis (e.g., proA, proB, and proC) decreased overall with increasing salinity, whereas proW increased significantly in S2, suggesting that under moderate salinity stress the rhizosphere microbiome may buffer osmotic pressure by enhancing proline-related processes, particularly transport/uptake linked to proW. Under higher salinity, proline biosynthesis (and/or related metabolism) may be suppressed, with a potential shift toward strategies that rely more heavily on ion-pumping systems and stress-maintenance functions. Notably, the increase in proW is more likely to reflect enhanced uptake/transport and/or local conditions that favor this strategy; however, the underlying mechanism requires further validation using functional annotations and metabolic evidence.

Overall, the halotolerance-gene results support the view that under high salinity, rhizosphere microorganisms may rely on a combined strategy of strengthened ion homeostasis and energy-efficient osmoprotection to sustain survival and functional activity. The enrichment of V/A-type ATPases and mnh/mrp in high-salinity samples, together with decreases in biosynthetic pathways for betaine, ectoine, and parts of proline metabolism, suggests that an adaptive mode characterized by “energy-intensive homeostasis maintenance” coupled with “energy-saving osmoprotection” may have been established under strong salinity stress. Specifically, intracellular ion balance and membrane potential were likely maintained via energy-dependent ion-pumping systems, whereas metabolic burdens may have been reduced by downregulating costly processes such as compatible-solute biosynthesis. Together, these adjustments may help preserve key metabolic functions and enhance community stability under salinity stress. These mechanistic lines of evidence strengthen our understanding of how the *Tamarix* rhizosphere microbiome adapts to salinized habitats and provide leads for future discovery of halotolerant functional microbes and potential targets for ecological restoration applications.

## 5 Conclusion

In this study, the structure and functional potential of the *Tamarix* rhizosphere microbiome across a salinity gradient were systematically characterized using shotgun metagenomics. As salinity increased, community diversity and richness decreased overall, and community composition shifted from bacterial dominance toward halophilic/halotolerant archaeal predominance. At the functional level, high salinity was associated with a shift from a “growth-expansion” to a “homeostasis-maintenance” strategy: functions related to genetic information processing (translation, transcription, and DNA replication/repair) and ion-homeostasis maintenance were enriched, whereas lipid metabolism, cell motility, and processes linked to secretion and collective behaviors were reduced. Meanwhile, the carbon-metabolic network appeared to undergo compensatory reconfiguration, with pathways such as glyoxylate and dicarboxylate metabolism upregulated under high salinity, potentially supporting energy demands and adjustments in carbon utilization. Analyses of salinity-adaptation genes further indicated that biosynthetic pathways for compatible solutes (betaine, ectoine, and proline) were generally downregulated, whereas the mnh/mrp Na^+^/H^+^ antiporter system and V/A-type ATPases were upregulated, suggesting a composite adaptive strategy centered on energy-dependent ion-homeostasis maintenance accompanied by energy-saving adjustments in osmoprotection pathways under strong salinity stress. Collectively, these findings suggest that the *Tamarix* rhizosphere microbiome enhances salinity tolerance through coordinated community restructuring and functional reprogramming, providing a data-driven basis for understanding plant–microbe interactions in salinized habitats and for developing microbially informed strategies for saline–alkaline soil restoration.

## Data Availability

The raw sequencing data have been deposited in the NCBI Sequence Read Archive (SRA) under BioProject PRJNA1370252.
